# Binary-blend fibber-based capture assay of circulating tumor cells for clinical diagnosis of colorectal cancer

**DOI:** 10.1186/s12951-017-0330-1

**Published:** 2018-01-16

**Authors:** Ai-Wei Lee, Fu-Xiang Lin, Po-Li Wei, Guo Jian-Wei, Jem-Kun Chen

**Affiliations:** 1 Department of Anatomy and Cell Biology, School of Medicine, College of Medicine, Taipei Medical University, Taipei, 110 Taiwan; 2 Department of Materials Science and Engineering, National Taiwan University of Science and Technology, 43, Sec. 4, Keelung Road, Taipei, 106 Taiwan, ROC; 3 Division of Colorectal Surgery, Department of Surgery, Cancer Research Center and Translational Laboratory, Taipei Medical University Hospital, Taipei Medical University, Taipei, Taiwan; 4 Division of Colorectal Surgery, Department of Surgery, Wan Fang Hospital, Taipei Medical University, Taipei, Taiwan; 5 Division of Colorectal Surgery, Department of Surgery, College of Medicine, Graduate Institute of Cancer Biology and Drug Discovery, Taipei Medical University, Taipei, Taiwan; 6 School of Chemical Engineering & Light Industry, Guangdong University of Technology, Guangzhou, China

**Keywords:** Electrospinning, Circulating tumour cell, Nylon-6, Colorectal cancer diagnosis

## Abstract

**Background:**

In addition to conventional approaches, detecting and characterizing CTCs in patient blood allows for early diagnosis of cancer metastasis.

**Methods:**

We blended poly(ethylene oxide) (PEO) into nylon-6 through electrospinning to generate a fibrous matbased circulating tumour cells (CTCs) assay. The contents of nylon-6 and PEO in the electrospun blend fibrous mats (EBFMs) were optimized to facilitate high cell-substrate affinity and low leukocyte adsorption.

**Results:**

Compared with the IsoFlux System, a commercial instrument for CTC detection, the CTC assay of EBFMs exhibited lower false positive readings and high sensitivity and selectivity with preclinical specimens. Furthermore, we examined the clinical diagnosis accuracy of colorectal cancer, using the CTC assay and compared the results with those identified through pathological analyses of biopsies from colonoscopies. Our positive expressions of colorectal cancer through CTC detection completely matched those recognized through the pathological analyses for the individuals having stage II, III, and IV colorectal cancer. Nevertheless, two in four individuals having stage I colorectal cancer, recognized through pathological analysis of biopsies from colonoscopies, exhibited positive expression of CTCs. Ten individuals were identified through pathological analysis as having no colorectal tumours. Nevertheless, two of these ten individuals exhibited positive expression of CTCs.

**Conclusions:**

Thus, in this population, the low cost EBFMs exhibited considerable capture efficiency for the non-invasive diagnosis of colorectal cancer.

## Background

Metastasis is the most common cause of cancer-related death in patients with solid tumours. A considerable body of evidence indicates that tumour cells are shed from primary and metastatic tumour masses at different stages of malignant progression. These breakaway circulating tumour cells (CTCs) [1] enter the bloodstream and travel to different tissues of the body as a crucial means of spreading cancer. The current gold standard for diagnosing tumour status requires invasive biopsy and pathological analysis. In addition to conventional approaches, detecting and characterizing CTCs in patient blood allows for early diagnosis of cancer metastasis. To address this unmet need, significant research endeavours, especially in the fields of chemistry, materials science, and bioengineering, have been devoted to developing CTC detection, isolation, and characterization technologies. Identifying CTCs in blood samples has, however, been technically challenging, because of the extremely low abundance (a few to hundreds per millilitre) of CTCs among a large number (10^9^ mL^−1^) of hematological cells.

A great number of separation systems have been developed, such as an antibody mediated immunoassay [2], size-based filtration method [3], fluorescence-activated cell sorting (FACS) [4], immunomagnetic separation [5, 6], dielectrophoresis force separation [7], and others, as summarized in previous reviews [8]. Among the popular methods, the immunomagnetic cell separation assay, which works by selectively labelling the CTCs with magnetic nanoparticles and using an external magnetic field to capture target cells, provides an effective solution for the translational clinical applications [9]. The immunomagnetic assay exhibits good sensitivity and specificity that arises from the cancer-specific antibody-antigen interactions. Therefore, some commercial instruments have been well-developed, such as the gold standard CellSearch system and IsoFlux system. These systems have exhibited outstanding cell capture efficiency (40–70%) when employed to isolate viable cancer cells from peripheral blood samples. However, sometimes a few leukocytes contaminate the CTC labelling system, resulting in false positive clinical diagnoses. In addition, positive expression of CTC detection alone is not enough to proceed with a diagnosis and treatment, limiting the clinical use of CTC detection. Most reports of CTC detection are focused on the high selectivity, specificity, and throughput of cell separation. Clinical diagnoses of cancer species by CTC detection are extremely rare [10].

**Fig. 1:**
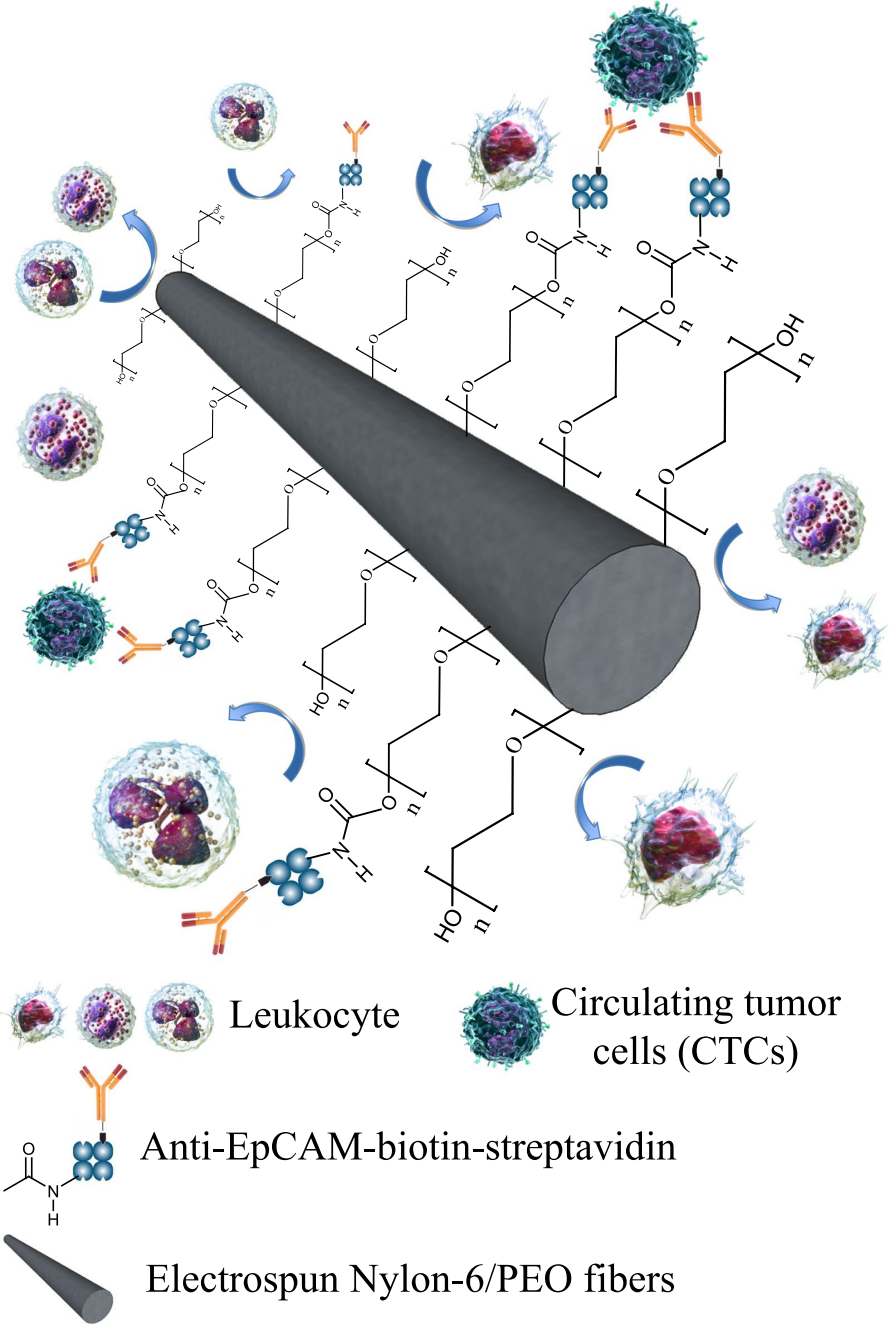
Schematic representation of CTC capture of EBFMs from blood specimens with repulsive ability against leukocyte adsorption

Approaches with engineered functional surfaces, using techniques such as chemically modified three dimensional micro/nano-structures, have been proposed to enhance the sensitivity of rare cell detection [11–13]. Significant research endeavours have been devoted to studying the interactions between live cells and nanostructured materials (e.g., nanofibres [14], nanotubes [15, 16], nanopillars [17, 18] that share similar dimensions with cellular surface components and extracellular matrix (ECM) scaffolds. Electrospinning is a simple and versatile nanofabrication technique [19, 20] for the preparation of ultra-long nanofibres with controllable diameters (from a few nanometres to several micrometres). A diversity of soluble and fusible polymers can be electrospun to form respective nanofibres from their precursor solutions. Electrospun nanofibres have the potential for use in a wide range of applications such as biocompatible/biodegradable scaffold matrices in tissue engineering [21, 22]. Other advantages of using electrospun nanofibres include (i) precise control over the dimensions and packing densities of the nanofibres; (ii) deposition of the nanofibres onto any given substrate (e.g., silicon, glass), using a well-established experimental setup; and (iii) the feasibility of engineering a variety of organic materials onto a cell capture substrate. In this study, we developed a simple method employing poly(ethylene oxide) (PEO) as a coupling reagent to immobilize streptavidin and, subsequently, anti-EpCAM antibody. PEO is a water-soluble, nontoxic, and nonimmunogenic polymer, and is among the most frequently used materials to reduce nonspecific protein adsorption [23]. To enhance the stability of the structure during CTC detection, nylon-6 was used as a biomaterial substrate for electrospinning because of its outstanding physicochemical properties [24, 25]; it is a relatively inert polymer in aqueous solution. The suppression of nonspecific cell adsorption at the solid–liquid interface of nylon-6/PEO blend electrospun fibres is expected for CTC detection. In this approach, we blended nylon-6 and PEO with formic acid to fabricate a highly rough surface through a one-step electrospinning process. After immobilizing anti-EpCAM on the surface, we used the electrospun blend fibrous mats (EBFMs) consisting of nylon-6 and PEO to capture CTCs from 7-mL specimens of blood (Fig. 1). Considering the geometric orientation of the nanostructures embedded in ECM scaffolds, electrospun nanofibres better mimic these horizontally oriented nanostructures, potentially leading to improved cell-substrate affinity. Therefore, pseudopodium could extend itself until the actin reassembles itself into a network. We chose to diagnose colorectal cancer by CTC detection in this work. Twenty-three individuals participated in a colorectal health check program, including colonoscopy biopsy with pathological analysis and the withdrawal of a 7-mL blood samples for CTC detection, to evaluate the efficiency of our CTC assay. Note that the blood samples from 23 individuals were not identified before CTC detection. These individuals were not patients, but felt colorectal discomfort, leading to their participations in this colorectal health check program. The diagnosis of colorectal cancer could be made by examining negative or positive expression of CTC for these individuals 6 h after the colonoscopy process. One week later, final recognition through pathological analysis revealed a diagnosis of the stage of colorectal cancer for these individuals.

## Experimental section

### Materials

Polycaprolactam (nylon 6, Polysciences) and PEO (Acros Organics), having average molecular weights (*M*_w_) of 18,000 and 1,000,000 g mol^−1^, respectively, were used as received. Red blood cell lysing buffer and hybri-max^TM^ fibrinogen from human plasma (50–70%, *M*_w_: 340 kDa), and sodium phosphate dibasic dihydrate were purchased from Sigma-Aldrich. Formic acid (Acros Organics) and NHS (Acros Organics, 98%) were used without further purification. EDC was purchased from Alfa-Aesar. Biotin anti-human CD326 (anti-EpCAM) and the FITC-streptavidin were purchased from BioLegeng (San Diego, CA). FITC-anti-EpCAM antibody and Cy5-biotin were purchased from Milli-Mark™ (Germany) and Click Chemistry Tools, respectively.

### Electrospun nylon-6/PEO fibrous mats

An 80% formic acid solution containing nylon-6 was stirred for 1 h. PBS was used to determine the optimal ratio of nylon-6 in the triple-blend electrospun fibrous mats. Binary mixtures of nylon-6 and PEO were formed in the formic acid solution at PEO-to-nylon-6 weight ratios of 10, 20, 30, 40, 50, and 60 wt%, giving samples denoted as N90/P10, N80/P20, N70/P30, N60/P40, N50/P50, and N40/P60, respectively. Formic acid solutions containing PEO were added dropwise into the nylon-6 solution and then the mixture was stirred for 1 h at room temperature. Concentrations of the mixture solutions for electrospinning were controlled at 22 wt%. For electrospinning, a syringe pump (KDS-100, KD Scientific) was fixed to a support that could be moved left and right at a speed of 7 m/min along a slipway to jet the nylon-6/PEO hybrid solution uniformly in the form of films on a rolled cylinder substrate. The metal needle tips of the syringes were connected to the positive electrode of a high voltage-power supply (YSTC Technology). The feeding rate of the polymer solutions was 0.3 mL/h. The applied voltage was 30 kV; the tip-to-collector distance was 15 cm. The EBFMs were collected through electrospinning onto the surface of a glass slip and dried at room temperature under vacuum for 24 h prior to subsequent characterization.

### Anti-EpCAM antibody immobilization

Poly(ethylene oxide) possesses carboxyl groups at the end of the polymer chain. Blending PEO within the nylon-6 matrix facilitates the affinity for inter-molecular hydrogen bonding between the amino group of nylon-6 and oxygen atoms of PEO. In addition, the presence of a hydrophobic domain of the alkyl groups within the blend results in the presentation of the carboxyl groups on the surface to immobilize the biotinylated anti-EpCAM (Fig. 1) [26]. Thereafter, EDC/NHS chemistry was employed for the immobilization of antibodies [27]. Briefly, the EBFMs were incubated in a PBS solution (pH 7.2–7.4) of EDC (2 mg/mL) and NHS (2 mg/mL) for 1 h at room temperature prior to modification. Streptavidin or FITC-streptavidin (127 μg/mL) was added to each sample, which was then stored at 4 °C overnight. Unreacted NHS, EDC, and streptavidin were washed away with the PBS for 10 min. The FITC-streptavidin-adhered EBFMs were subsequently observed using a fluorescence microscope (FL Color Imaging System, AMF 4300, Life Technologies). The streptavidin-modified fibrous mats were incubated in biotinylated anti-EpCAM antibody (5 μg/mL) to form the substrate for the cell-capture experiments. The samples were gently washed three times with PBS to remove any non-immobilized antibodies.

### Surface chemical characterization of modified blend fibrous mats

The surface morphologies of the electrospun nanofibres were characterized through field emission scanning electron microscopy (FE-SEM), using a JSM 6500F instrument operated at 15–20 kV. Fibre diameters were determined using Image-J image processing software. For each electrospun mat, at least 100 fibres were considered from three different images to calculate the average diameter. The roughness of the triple-blend fibrous mats was examined using an ultra-precision benchtop 3D optical profiler (UPBOP, Talysurf CCI LITE, Taylor Hobson). The distributions of the biomacromolecules on the fibre surfaces were investigated through laser scanning confocal microscopy (LSCM), using a Leica TCS SP5 confocal spectral microscope imaging system featuring a 100-mW Ar blue laser operated at 494 nm. A red fluorescent antibody (Cy5-Biotin, Click Chemistry Tools) was employed to determine the distribution of the biotinylated anti-EpCAM antibodies on the EBFMs. The streptavidin-modified EBFMs were placed in a Cy5-Biotin solution (5 μg/mL) at room temperature. The Cy5-Biotin-adhered EBFMs were subsequently observed through LSCM at 651 nm. The grafting density of anti-EpCAM could be estimated by the emission of Cy5-biotin at 651 nm of wavelength.

### Non-biofouling properties of anti-EpCAM-modified EBFMs

Leukocytes were employed to examine the non-biofouling properties arising from adsorption upon the anti-EpCAM-modified EBFMs as well as fibrinogen [28, 29]. Leukocytes generally generate a significant obstruction for CTC absorption and recognition. A drop of a solution of leukocytes (400 μL) isolated from peripheral blood (10 mL) was placed onto the anti-EpCAM-modified EBFMs, and then the system was incubated for 360 min at room temperature. After the leukocytes had been stained with hematoxylin, the samples were observed by optical microscopy (OM, Olympus, BX 43). Total number of leukocytes was counted to calculate the ratio of the adhesive leukocytes. Each final result was the average of three determinations.

### Colorectal tumour cell adhesion in preclinical assay

The human colorectal cancer cell lines DLD-1, HCT-116, and HT-29 were purchased from the American Type Culture Collection (ATCC, Manassas, VA, USA). Cell lines were cultured in RPMI-1640 medium (Gibco-Invitrogen, Carlsbad, CA) with 10% foetal bovine serum (Gibco-Invitrogen), glutamine, penicillin, and streptomycin and maintained in a humidified atmosphere containing 5% CO_2_ at 37 °C. Anti-EpCAM-modified EBFMs were placed in the wells of a 24-well plate (Corning). Cell lines were incubated in PBS containing FITC-anti-EpCAM antibody (5 μg/mL) in the dark for 1 h. Various counts of tumour cell lines were diluted with PBS (2 mL) prior to use. Peripheral blood samples (5 mL) were collected in tubes containing heparin anticoagulant. The PBS (2 mL) possessing various counts of tumour cell lines was mixed with the peripheral blood samples (5 mL), drawn from healthy donors that no other pathologies or negative for colon cancer, as specimens for CTC capture. The protocol for CTC capture by EBFMs from peripheral blood samples was as follows. The specimens (7 mL) were centrifuged at 3000 rpm for 15 min. The buffy coat was collected and mixed with RBC lysis buffer at 1200 rpm for 6 min to remove most of the erythrocytes. The process was repeated four times to collect the buffy coat, which was then incubated in PBS containing FITC-anti-EpCAM or Alexa594-conjugated anti-Ck8+18 (5 μg/mL) in the dark for 1 h. Droplets of a mixture (300 μL) of PBS and buffy coat were placed on the anti-EpCAM-modified EBFMs (1 × 1 cm^2^) after incubation for 3 h. Thereafter, the fibrous mats were gently washed three times with PBS to remove any uncaught cells and incubated in 4% paraformaldehyde for 10 min to fix the cells adhering to the surface. After staining with hematoxylin, the samples were imaged using OM (Olympus, BX 43) and the CTCs on the anti-EpCAM-modified EBFMs (1 × 1 cm^2^) were counted using software (Image-Pro Plus V7) by scanning all areas of the EBFMs.

### Clinical diagnosis of colorectal cancer by CTC detection

An IsoFlux system (Fluxion Biosciences Inc., South San Francisco, CA, United States) was included to capture CTCs as a clinical assay for comparison with CTC assay of EBFMs. The commercial IsoFlux System, an instrument currently used to detect CTCs in hospitals, is registered as a Class I medical device with the US FDA. According to a previous study, the sensitivity of the IsoFlux system for CTC identification is higher than that of the gold standard CellSearch system [30–32]. The protocols for the IsoFlux system for CTC identification were as follows:

1. A 7–10 mL peripheral blood specimen is injected into the instrument.
2. Magnetic beads are modified by cytokeratin antibodies to mix with the specimen. These marker-modified magnetic beads attach to CTCs specifically in the solution.
3. The magnetic bead-attached CTCs are picked up from the specimens one by one onto a platform (1 × 1 cm^2^) for CTC identification.
4. Fluorescence agencies including cytokeratin, CD45 (leukocyte marker), and nucleus (DAPI) marker are exploited to stain all cells on the platform. The system software is used to record fluorescence intensity and species by scanning all regions of the platform to identify all cells as CTCs or leukocytes and obtain CTC counts. Because of the large amount of leukocytes, some leukocytes inevitably appear on the platform. Seven subjects participated in the program; peripheral blood from each subject was collected and equally divided into specimens for the CTC capture assay and IsoFlux system, respectively.

Peripheral blood samples were collected in tubes containing heparin anticoagulant from 23 individuals, who also underwent examination through invasive colonoscopy biopsies followed by pathological analysis under a clinical protocol entitled “Study of detection circulating tumour cell on micro-nano surface” which was approved by the Institutional Review Board (IRB) of Taipei Medical University (201311008). Blood was obtained from patients or healthy donors following written informed consent, which was also approved by the IRB committee. Following the protocol of CTC capture with EBFMs, the CTC images and counts on the fibrous mats were obtained by fluorescence microscopy (FL color Imaging System, Life Technologies AMF 4300) and software (Image-Pro Plus V7) by scanning all areas of the EBFMs. For each specimen, the negative and positive expressions of CTC detection were compared with the results from the pathological analysis of biopsies from the colonoscopy and the IsoFlux system to evaluate the accuracy of the method. All methods were carried out in accordance with relevant guidelines and regulations. All specimens were sampled from patients after giving informed consent. All data are presented from observations made using three biological repeats. Three independent experiments and statistical test used were exploited to obtain all results. Results were reported as mean ± standard deviation (SD), and a minimum of triplicates were performed in each experiment. Statistical analysis was tested by T test for two groups. Statistically significant difference was considered at p < 0.05, highly significance was p < 0.01. All statistical analyses were performed by SPSS 19.0 (IBM Corporation, USA) with the following formula:

$${\text{T}} = \frac{{\overline{{x_{1} }} - \overline{{x_{2} }} }}{{\sqrt {\frac{{s_{1}^{2} }}{{n_{1} }} + \frac{{s_{2}^{2} }}{{n_{2} }}} }}$$

where x_1_ and x_2_ represent the mean of first or second set of values; s_1_ and s_2_ represent the standard deviation of first or second set of values; n_1_ and n_2_ represent total number of values in first or second set. p value examination is referred to the T distribution table.

## Results and discussion

### Surface characterization

**Fig. 2:**
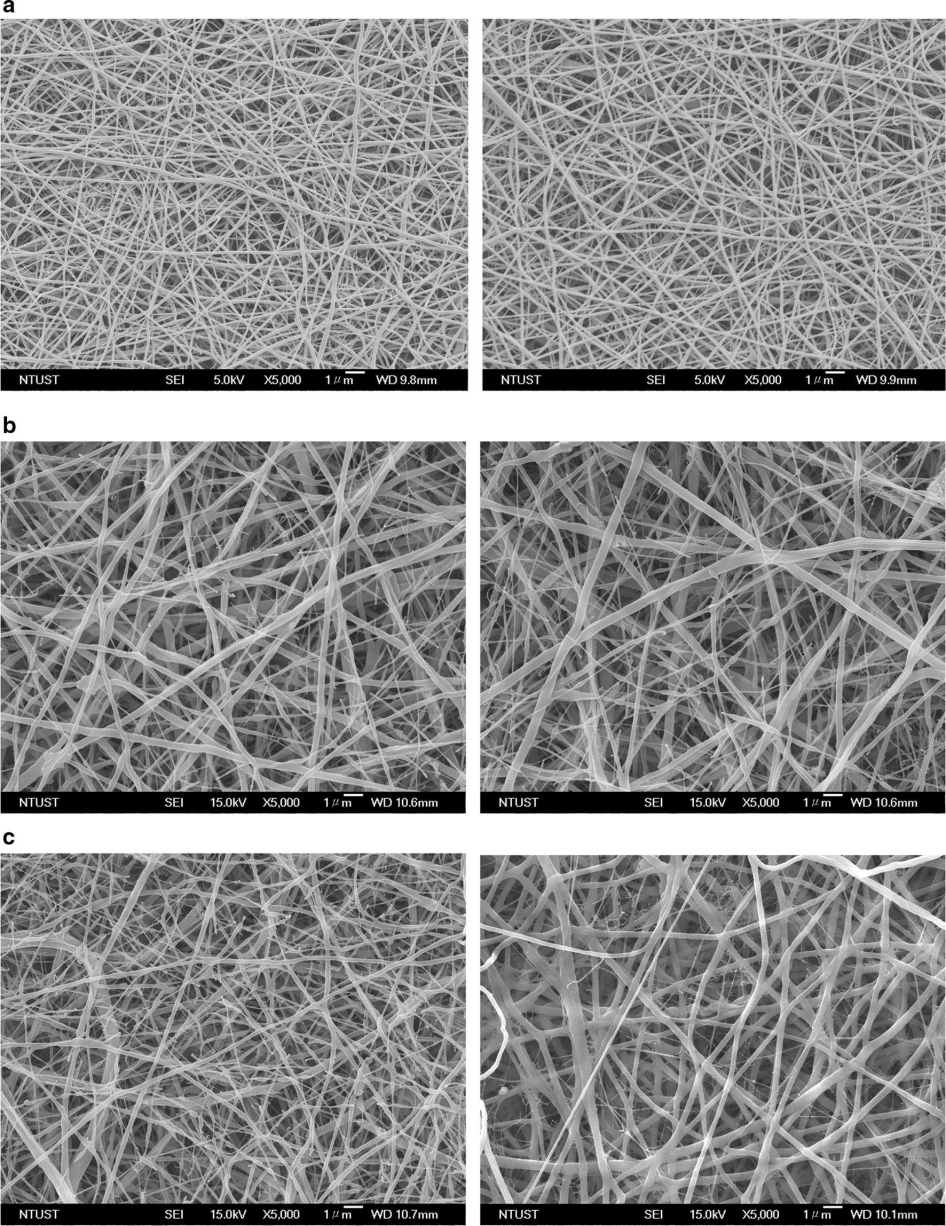
Representative SEM micrographs of electrospun nylon-6/PEO fibres: **a** N90/P10, **b** N80/P20, **c** N70/P30

**Fig. 3:**
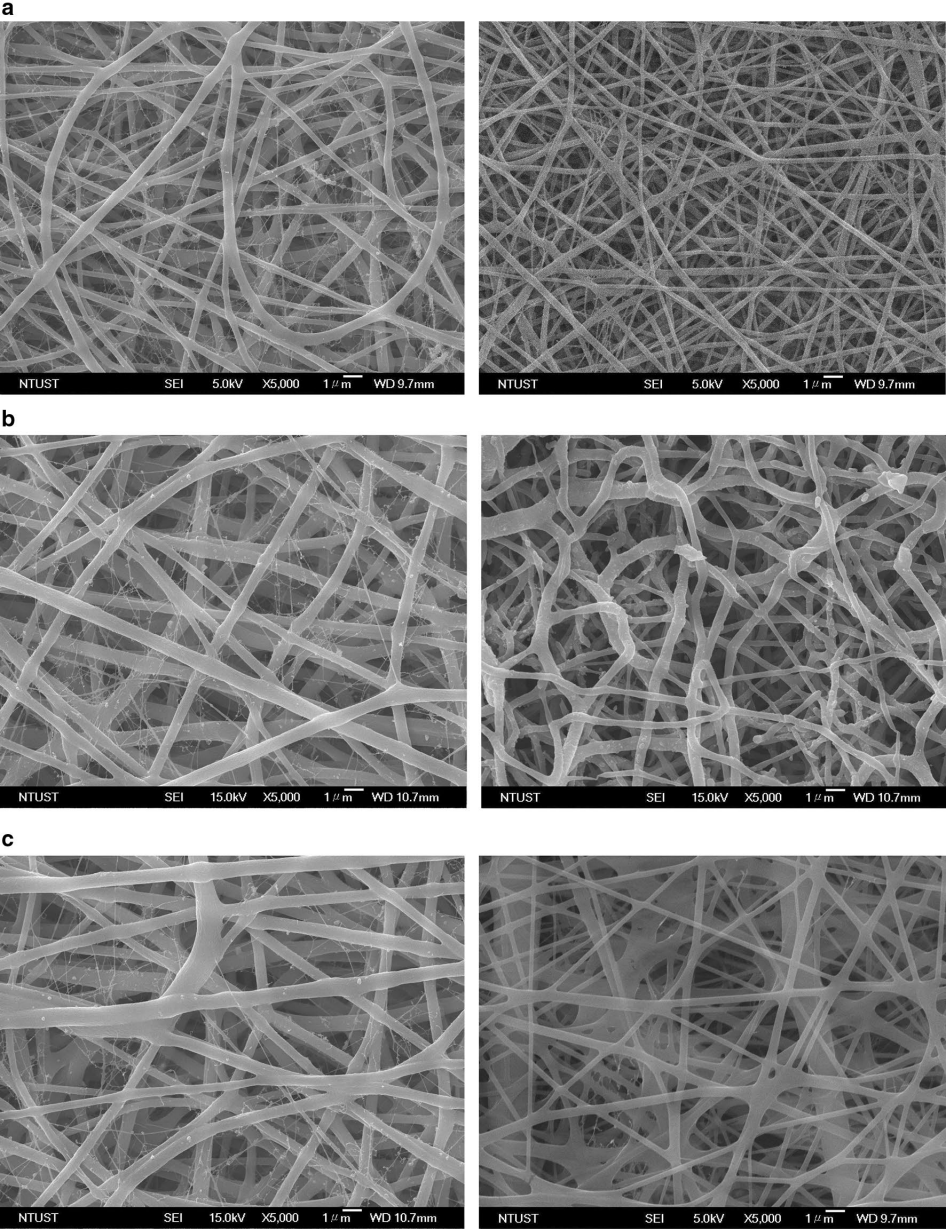
Representative SEM micrographs of electrospun nylon-6/PEO fibres: **a** N60/P40, **b** N50/P50, and **c** N40P60

Figures 2 and 3 displays SEM images and fibre diameter distributions of the EBFMs N90/P10, N80/P20, N70/P30, N60/P40, N50/P50, and N40/P60 before and after incubation for 3 h in PBS. When a pure nylon-6 solution is used for electrospinning, a spider-net structure among fibres forms because of the interaction between nylon-6 and formic acid [33]. The fractions of PEO in the nylon-6 solution had a marked effect on the fibre diameter and morphology. The fibre diameter of the electrospun N90/P10 was 295 ± 76 nm (Fig. 2a, left). Increasing the fraction of PEO relative to nylon-6 resulted in larger fibre diameters, suggesting that the high-molecular-weight PEO substantially extended the diameter of the EBFM fibres (Fig. 2b, left). After further increasing the fraction of PEO relative to nylon-6 (sample N70/P30), the fibre diameters exhibited a broad distribution (Fig. 2c, left). The diameter distribution for N60/P40 could be divided into two main sizes: 396 ± 97 and 1531 ± 274 nm (Fig. 3a). For the N50/P50 and N40/P60 blend, the fibre diameters were 1352 ± 196 nm without significant irregularity in the diameter distributions (Fig. 3b, left and c, left), indicating that the high-molecular-weight PEO predominately determined the fibre diameter. Since dimensions of surface structures similar to that of pseudopodia facilitate the attachment of tumour cells on the surface, degradation of fibres usually result in a decrease in roughness [34]. We observed the morphology of the EBFMs (within an area of 200 × 200 μm^2^) after incubation in PBS for 3 h corresponding to the CTC capture time. With the exception of N40/P60, we did not observe significant changes in their morphology after incubation in PBS for 3 h, suggesting that morphology would be retained almost completely during the CTC capture. For N40/P60, obvious degradation occurred resulting in the extension of diameter and collapse of fibre structure. Therefore, the fractions of PEO relative to nylon-6 in the blend solutions for electrospinning were set at 50% to achieve non-biofouling properties and anti-EpCAM binding as rich as possible, with high stable fibre structure during CTC capture.

**Fig. 4:**
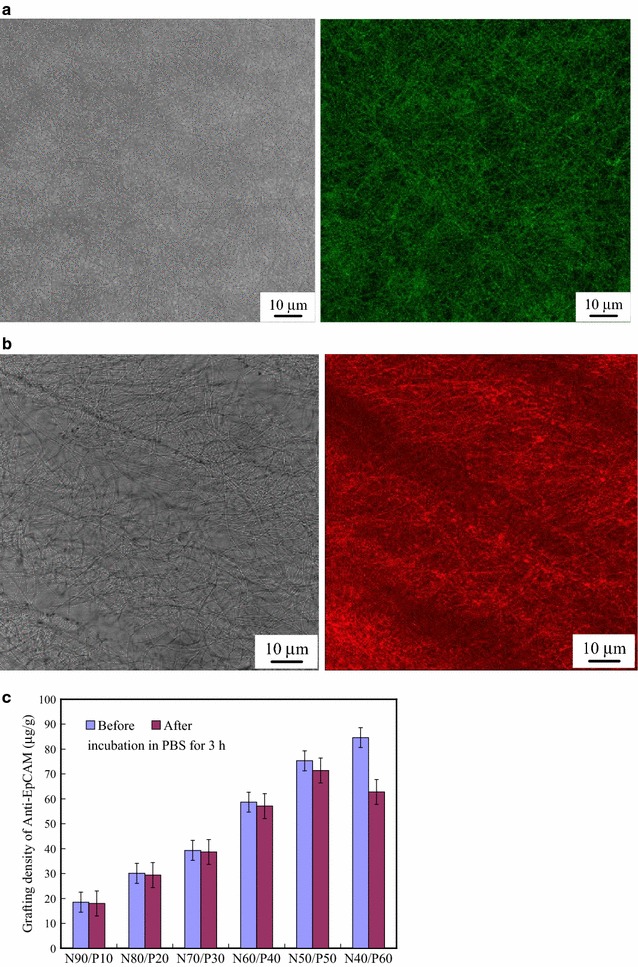
LSCM images of **a** FITC-streptavidin-modified N50/P50 and **b** the sample in (**a**) after the immunosorbent reaction with Cy5-biotin. **c** Grafting density of anti-EpCAM on the N50/P50 before and after incubation in PBS for 3 h (p value < 0.04)

We grafted FITC-streptavidin and Cy5-biotin onto the N50/P50 surface to observe their distribution by LSCM and to overcome the non-fluorescence of streptavidin and biotin anti-EpCAM. The results represented the efficiency of the immunosorbent reaction between streptavidin and anti-EpCAM. Figure 4 displays the LSCM images of N50/P50, FITC-streptavidin, and Cy5-biotin/FITC-streptavidin-grafted N50/P50. Prior to grafting FITC-streptavidin on N50/P50, the EBFM did not exhibit fluorescence completely on the surface. When FITC-streptavidin was grafted on the surface, fluorescence was evident in the LSCM image (Fig. 4a). Since the resolution of LSCM imaging is approximately 1 μm, fibres having diameters of less than 1 μm could be seen clearly. From comparison with the OM image, the streptavidin grafting appeared to be uniform on the fibres. The red fluorescence of Cy5-biotin was clearly evident after the immunosorbent reaction between FITC-streptavidin and Cy5-biotin (Fig. 4b). Figure 4c displays the grafting density of anti-EpCAM on the N90/P10, N80/P20, N70/P30, N60/P40, N50/P50, and N40/P60, respectively. All p values of the results are less than 0.04. The grafting density of anti-EpCAM increased with PEO content before PBS incubation for 3 h; on the other hand, increasing the PEO content facilitated the grafting of anti-EpCAM on the surface. The anti-EpCAM grafts decreased significantly because of the degradation, consistent with the SEM images. Therefore, N50/P50 could be regarded as an optimal surface.

### Non-biofouling properties

**Fig. 5:**
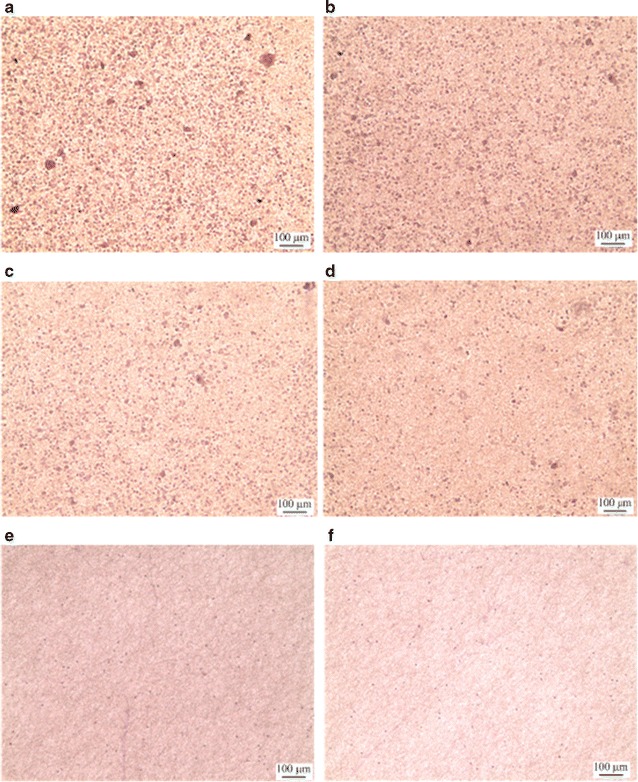
**a**–**e** Representative photographs of adherent leukocytes on antibody-immobilized **a** N90/P10, **b** N80/P20, **c** N70/P30, **d** N60/P40, **e** N50/P50, and **f** N40P60

A large number of leukocytes contaminate the samples during the process of staining CTCs with fluorescence to identify CTCs, resulting in false positive diagnosis. To examine the adsorption of leukocytes, the anti-EpCAM-modified EBFMs were incubated with leukocytes (ca. 8 × 10^6^). Therefore, a decrease in leukocytes on the surface is important for eliminating the false positive result. Figure 5 displays the results of coagulation experiments with the leukocytes after incubation for 3 h. Figure 5a–e present typical OM images of the leukocytes adhered onto the anti-EpCAM-modified N90/P10, N80/P20, N70/P30, N60/P40, N50/P50, and N40/P60 surfaces, respectively. The purple spots in these OM images represent fouled leukocytes, stained with hematoxylin, on the anti-EpCAM-modified EBFMs. Figure 5f displays the statistical ratios of adhesive leukocytes on the N90/P10, N80/P20, N70/P30, N60/P40, N50/P50, and N40/P60, respectively. All p values of the results are less than 0.05. The number of adsorbed leukocytes decreased upon increasing the PEO content in the anti-EpCAM-modified EBFMs. Few leukocytes were present on the N50/P50 and N40/P60 fibrous mat, because of its high content of PEO. We estimated the ratio of adhesive leukocytes obtained by counting, followed by unit conversion. The ratios of adhesive leukocytes for N90/P10, N80/P20, N70/P30, N60/P40, N50/P50, and N40/P60 were 8.613 ± 0.112, 3.625 ± 0.0743, 0.184 ± 0.0083, 0.0214 ± 0.0029, 0.007 ± 0.0003, and 0.002 ± 0.0001%, respectively. All p values of the results are less than 0.05. Note that the attached leukocytes were not dispersed on the surface uniformly. Some defects in the fibrous mats facilitated leukocyte attachment. Once a leukocyte attaches onto the defect region of the surface, the leukocyte changes the non-biofouling properties of the defect region resulting in more leukocytes attaching to the region. According to our observation, a few leukocytes assembling predominately determined the 0.007 and 0.002% leukocyte attachment ratio on 1 cm^2^ for N50/P50 and N40/P60. Therefore, we selected N50/P50 for CTC capture tests from peripheral blood specimens, because of its appropriate fibre structure, resistance to degradation, antibody-binding ability, and non-biofouling properties.

### Colorectal tumour cell capture from peripheral blood specimens

**Fig. 6:**
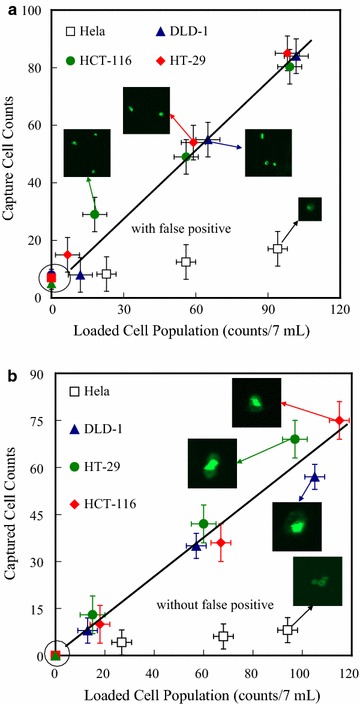
Counts of colorectal cancer cells (DLD-1, HCT-116, HT-29) captured by **a** IsoFlux system and **b** the N50/S25/A25 fibrous mats (1 × 1 cm^2^) with anti-EpCAM grafts, plotted with respect to the counts of cells loaded in 7-mL blood specimens

We performed tumour cell capture experiments to examine the possibility of capturing CTCs, using our anti-EpCAM-modified EBFMs. EpCAM is a 40-kDa monomeric membrane glycoprotein that is overexpressed in most carcinomas; it is used widely as a target molecule in existing CTC detection methods because of its low expression in normal blood cells. Nevertheless, the level of expression of EpCAM in cancer cell lines can be variable [26]. Therefore, we used immunocytochemistry to investigate the EpCAM expression of three colon cancer cell lines prior to performing clinical diagnoses. A few colorectal cancer cells (< 120 counts), including DLD-1, HCT-116, and HT-29, were loaded into samples (7 mL) of blood, respectively. A blank experiment, a clean sample of peripheral blood without the cells, was also included to examine the accuracy. All of the captured colorectal cancer cells on the EBFMs over an area of 1 × 1 cm^2^ were recorded to evaluate the cell capturing efficiency. In addition, we also exploited the IsoFlux system, a commercial instrument, to evaluate the efficiency for comparison. Figure 6a displays the relationship between the loaded and captured colorectal cancer cells from the peripheral blood specimens by the IsoFlux system. The four kinds of colorectal cancer cell lines, DLD-1, HCT-116, HT-29 and Hela, all exhibited positive expression by the IsoFlux system. We observed approximately linear increases in the number of captured cells upon increasing the number of loaded cells, indicating the stable cell capture ability of the IsoFlux system. The degree of cell capture accuracy was statistically increased with the loaded cell population in the specimen expect Hela cell line. A false positive appeared in the blank experiment, indicating that there were contaminated cells due to a large number of leukocytes. Note that the CTCs were counted using software (Image-Pro Plus V7) by scanning all areas instead of artificial identification. For the positive expression and stable cell capture ability of the EBFMs, remarkably, suggesting stable capture efficiency (Fig. 6b). 4, 6, and 8 counts of Hela cell lines from the 7-mL blood samples loaded with 27, 68, 94 counts were captured, indicating high capture selectivity as well. No false positive was observed in the blank experiment. Thus, CTCs could be captured by the EBFMs in the preclinical assay at extremely low abundance (a few to hundreds per millilitre) of CTCs and identified readily among the large number of leukocytes. The capture ratio by the IsoFlux System ranged between 66.7 and 70.6%, similar to that of the EBFMs.

**Fig. 7:**
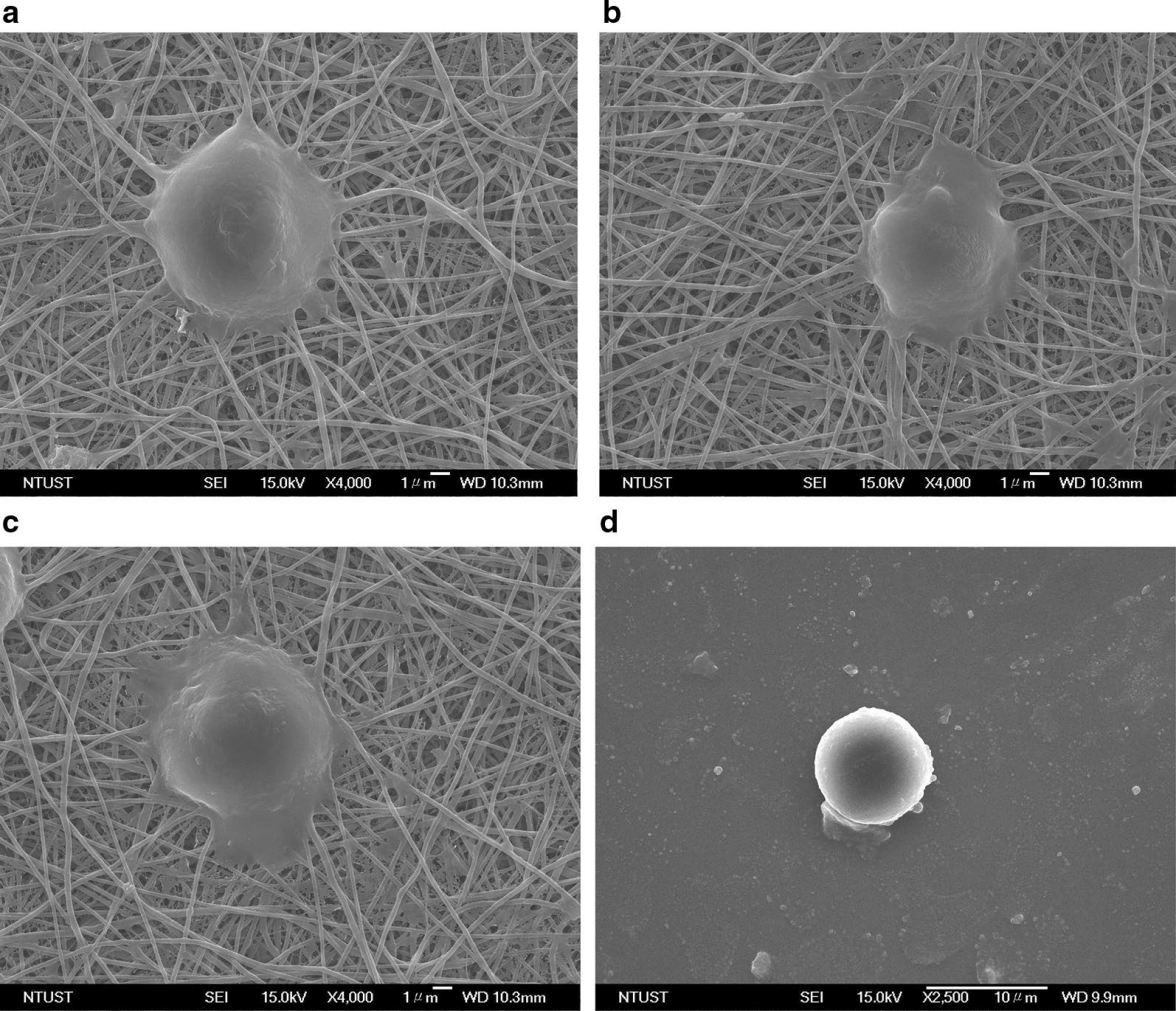
SEM images of captured colorectal cancer cells: **a** DLD-1, **b** HCT-116, and **c** HT-29 on the N50/P50 fibrous mats, and** d** DLD-1 on a glass surface with anti-EpCAM grafts

**Fig. 8:**
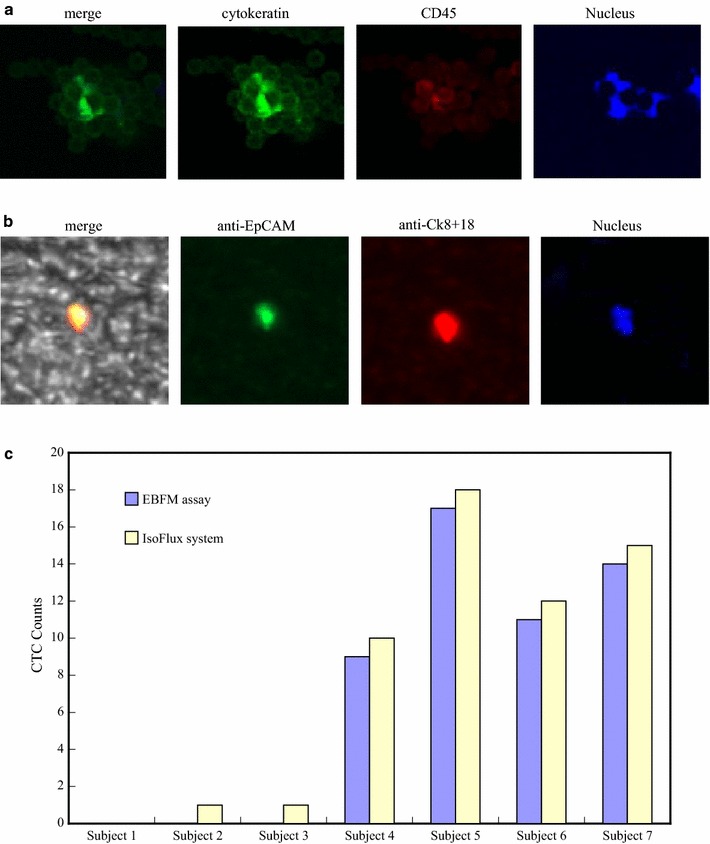
**a** Fluorescence images on the platform of the IsoFlux System, resulting from cytokeratin (green fluorescence), leukocyte (red fluorescence), and nucleus marker staining, and phase contrast image superimposed on the fluorescence image for an example of CTC recognition. **b** Fluorescence images on the EBFM assay, resulted from the FITC-conjugated anti-EpCAM (green fluorescence), Alexa594-conjugated anti-Ck8+18 (red fluorescence), and leukocyte marker (CD45) staining, and phase contrast image superimposed on the fluorescence image for an example of CTC recognition. **c** CTC counts for seven subjects, obtained using IsoFlux system, N50/P50 fibrous mats with anti-EpCAM grafts

Figure 7a–c shows typical SEM images of target cells captured on the anti-EpCAM-modified N50/P50. Single target cells of DLD-1, HCT-116, and HT-29, with fully outspread pseudopodia, attached to the surface of anti-EpCAM-modified N50/P50 are clearly evident in Fig. 7a–c, respectively. Compared with the DLD-1 attached to a glass surface (Fig. 7d), the CTCs substantially attached onto the fibrous structure with fully outspread pseudopodia. The fibre structure after CTC capture mostly remained intact without significant degradation. The diameters of the pseudopodia and the fibres are well matched to ensure sufficient contact and efficient adhesive force, resulting in improved cell-substrate affinity [11, 17]. This observation is consistent with the results obtained individually under the various experimental conditions, suggesting that the horizontal packing of the EBFMs might have been responsible for the improved cell capture yields [11]. CTCs usually detached from the fibrous mats without pseudopodium extension. Our results revealed that good affinity toward colorectal tumour cells could be obtained after modification and antibody immobilization of N50/P50. Thus, in these specimens, the static fibrous mats exhibited considerable capture efficiencies. Furthermore, diagnosis accuracy of the CTC assay of EBFM was evaluated with seven known specimens, including three negative and five positive subjects of colorectal cancer, as well as by using the IsoFlux system. The platform area of the CTC assay of EBFM and IsoFlux System is ca. 1 cm^2^, indicating that artificially identifying cells all over the platform with fluorescence microscopy may take ca. 8 h for each specimen. A fluorescence scanning system (Image-Pro Plus V7) was used to scan all areas of samples to identify each cell with cytokeratin, leukocyte, and nucleus marker staining all over the platforms automatically to reduce the time required for CTC recognition. Figure 8a displays fluorescence images on the platform of the IsoFlux System, resulting from cytokeratin (green fluorescence), leukocyte (red fluorescence), and nucleus marker (blue fluorescence) staining, and a phase contrast image superimposed on the fluorescence image for an example of CTC recognition by the fluorescence scanning system. The cytokeratin marker-stained cells could be defined generally as CTCs. However, leukocytes surrounding the CTCs caused contamination, which generated a fluorescent halo that may have influenced the CTC recognition by the fluorescence scanning system and facilitated a false positive. In addition, the assembling leukocytes also might have partially blocked the nucleus marker. As a result, leukocytes, based on CD45 staining, did not express the nucleus marker. Figure 8b displays fluorescence images on the EBFM assay, resulted from FITC-conjugated anti-EpCAM (green fluorescence), Alexa594-conjugated anti-Ck8+18 (red fluorescence), nucleus marker (DAPI) staining, and phase contrast images superimposed on the fluorescence image. The stained cells that exhibited positive expression of EpCAM and CK8+18, and that were morphologically intact could be defined as CTCs, as shown in Fig. 8b. The fluorescence focused on the CTCs predominately without the halo of contaminated leukocytes nearby, which presumably reduced the false expression identified by the fluorescence scanning system. Figure 8c displays the CTC counts captured by the IsoFlux system and the CTC assay of EBFM from seven specimens including three healthy subjects (1, 2, and 3) and four colorectal cancer patients (4, 5, 6, and 7). Our negative expressions of colorectal cancer through the CTC assay of EBFMs matched with the expression of subject 1, subject 2, and subject 3. However, healthy subject 2 and 3 were diagnosed with positive expression, so-called false positive, due to the contamination of leukocytes through the IsoFlux system. Therefore, the diagnosis results of IsoFlux need to be checked by manual observation, which is not popular in the clinical setting. In addition, similar CTC counts were detected in 7.5 mL of blood from patient subjects 4, 5, 6, and 7 and diagnosed as positive expression through the CTC assay of EBFMs and the IsoFlux System. Positive expressions of colorectal cancer through the CTC assay of the EBFMs completely matched with the expression of these specimens. Therefore, the CTC assay of EBFMs, with extremely low cost, has potential for use in rapid clinical diagnosis of colorectal cancer.

**Fig. 9:**
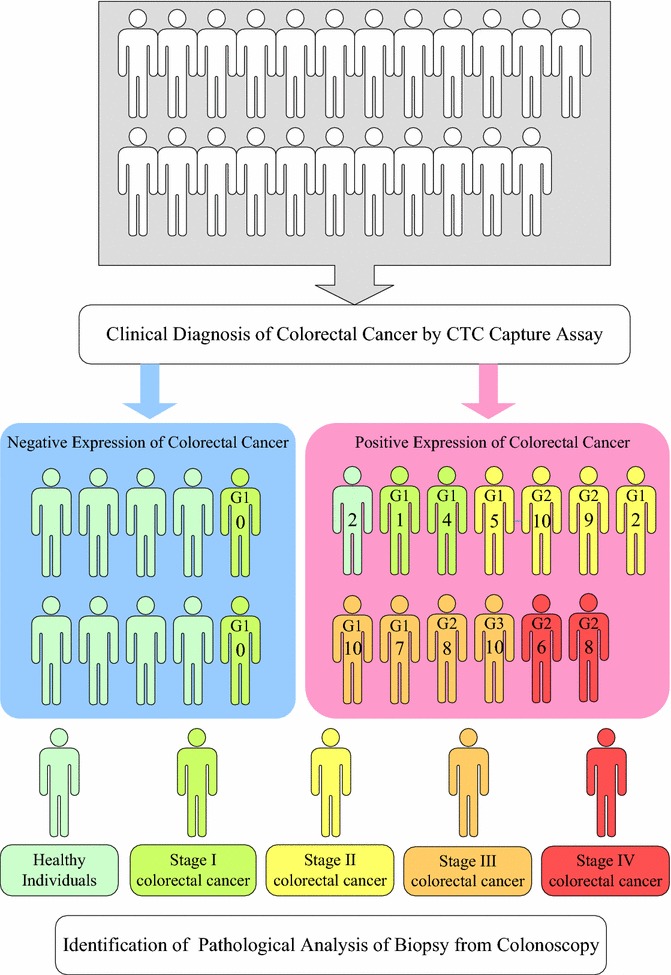
Clinical diagnoses for 23 individuals, performed using both CTC detection with the antibody-immobilized N50/P50 and pathological analysis of biopsies from colonoscopies. The number in each individual marked positive expression (red colour) of colorectal cancer represents the CTC counts on the fibrous mat-based assay. G1 and G4 represent Grade 1 and Grade 4 that means the cancer looks much like normal colorectal tissue and very abnormal, respectively. G2 and G3 represent Grades 2 and 3 that fall somewhere in between

Next, we employed in vitro CTC capture experiments with the CTC assay of EBFM for clinical diagnosis. Twenty-three individuals were examined through invasive colonoscopy biopsies, followed by pathological analysis; blood (7 mL) was also drawn for the CTC capture assay. In general, pathological analysis of biopsies from colonoscopies takes about 1 week to recognize colorectal cancer at different stages of malignant progression. In contrast, detecting CTCs from a peripheral blood specimen requires only approximately 6 h and is much shorter than the time necessary for pathological analysis. Therefore, the cancer status of the individuals who gave these blood specimens for CTC detection was unknown until the results from the pathological analyses had been obtained. Figure 9 summarizes the results of the diagnoses of colorectal cancer for all participators, as determined through CTC capture and pathological analysis of biopsies from colonoscopies. Note that the diagnosis of colorectal cancer through CTC capture alone was dependent on the positive and negative expression of colorectal cancer. The stages of colorectal cancer are recognized through pathological analysis, because CTC counts may not be related significantly to the cancer stage. Our positive expression of colorectal cancer through CTC detection matched those recognized through the pathological analyses for the individuals having stage II, III, and IV colorectal cancer. Nevertheless, only two in four individuals having stage I colorectal cancer, recognized through pathological analysis of biopsies from colonoscopies, exhibited positive expressions of CTCs, suggesting that metastasis of stage I colorectal cancer may be temporarily lower [35]. Ten individuals were identified through pathological analysis as having no tumours. Nevertheless, two of these ten individuals exhibited positive expression of CTCs, possibly identifying them as having other cancers with metastasis. Note that anti-EpCAM is not only specific for colorectal cancer but also other cancers, such as breast and prostate cancer, since there is a cross-talk [36]. False positive results are unavoidable in clinical diagnosis because there are no antibodies specific for just one kind of cancer that have been identified. Thus, in this population, the possibility of colorectal cancer diagnosis through the CTC assay of EBFM was considerable. Pathological analyses of biopsies from colonoscopies generally require a doctor, a nurse, and an anaesthetist, and at least 1 week for identification. CTC detection using static fibrous mats significantly decreases the required manpower and time, providing great potential for rapid cancer screening.

## Conclusions

We have developed a CTC capture platform based on a high-affinity cell enrichment assay, employing electrospun nanofibres deposited on a substrate and coated with a cell-capture agent. A non-invasive diagnosis approach with low cost of nylon-6/PEO fibrous mats was developed to facilitate the diagnosis of colorectal cancer, which could motivate people to participate in colorectal health checks more frequently. For colorectal cancer, the cure rate decreases significantly with cancer stage because of cancer cell metastasis. Although the diagnostic accuracy of stage I colorectal cancer was insufficient, it also indicates lower risk of metastasis in stage I colorectal cancer. In addition, false positive diagnosis of colorectal cancer also indicates the high risk of metastasis in other cancers, providing extra information for the patient. The fibrous assay has been used clinically in circulating cells irrespective of the sort of cancer suffered the subjects. Once the antibody with high selectivity for particular sort of cancer has been developed, it could be further universalized to detect particular sort of cancer. Therefore, this platform based on electrospun fibrous mats has high potential applications in early non-invasive diagnosis and longitudinal monitoring of cancer in clinics.
